# Case Report: Balancing immune responses – multiple sclerosis disease exacerbation under BRAF/MEK treatment for malignant melanoma

**DOI:** 10.3389/fonc.2023.1303141

**Published:** 2023-11-24

**Authors:** Katrin Pape, Maria Protopapa, Muriel Schraad, Falk Steffen, Frauke Zipp, Stefan Bittner

**Affiliations:** Department of Neurology, Focus Program Translational Neuroscience (FTN) and Immunotherapy (FZI), Rhine-Main Neuroscience Network (rmn2), University Medical Center of the Johannes Gutenberg University Mainz, Mainz, Germany

**Keywords:** malignant melanoma, BRAF/MEK treatment, multiple sclerosis, B cell depletion, ocrelizumab, multi-color flow cytometry, individualized therapy

## Abstract

**Background:**

Combination treatment with BRAF/MEK inhibitors favorably impact progression-free survival in malignant melanoma. However, it may cause paradoxical activation of the MAPK/ERK pathway in immune cells without BRAF mutation, which may lead to over activation of the immune system, especially in patients with pre-existing autoimmune conditions. In this case report, treatment of malignant melanoma with BRAF/MEK inhibitors was associated with radiological disease exacerbation of pre-existing multiple sclerosis (MS).

**Case presentation:**

A 47-year-old patient with pre-existing MS was diagnosed with malignant melanoma in June 2020. Anti-tumor treatment was initiated with a combination therapy of BRAF inhibitor dabrafenib and MEK inhibitor trametinib. In February 2022, the patient presented at our neurological clinic after routine MRI revealed exacerbation of radiological MS disease activity with ten new and gadolinium-enhancing lesions, and concomitant high levels of neurofilament light chain (NfL) in serum, a marker for axonal damage. In-depth analysis of immune cells in both peripheral blood and cerebrospinal fluid was performed by multi-color flow cytometry. After treatment with the B cell-depleting antibody ocrelizumab, MS disease stability was obtained and anti-tumor medication could be continued.

**Conclusions:**

Immunomodulatory treatment in cancer patients is highly effective from an oncological point of view, but may be associated with autoimmune side effects. This is of special importance in patients with pre-existing autoimmune diseases, as reflected by our case of MS disease reactivation under treatment with BRAF/MEK inhibitors. In our case, sequential modulation of immune cell subsets by B cell depletion, associated with marked shifts in B and T cell subsets, allowed for stabilization of disease and continuation of anti-tumor treatment.

## Introduction

**Figure 1:**
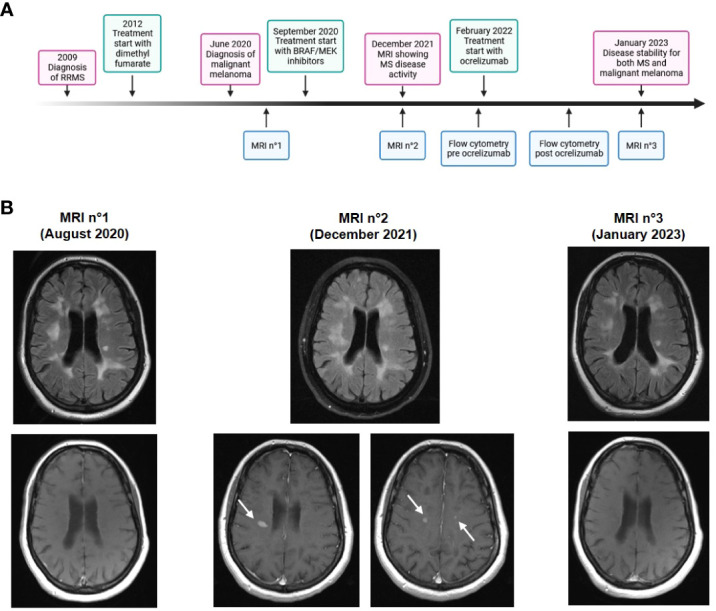
Time course and cerebral imaging. **(A)** Timeline showing clinical, diagnostic and therapeutic developments. **(B)** Cerebral MRI scans at different time points. Upper panels: T2 fluid-attenuated inversion recovery (FLAIR)-weighted image, lower panels: T1-weighted image with contrast enhancement. MRI n°2 shows multiple new and contrast-enhancing lesions (arrows).

Immune modulation has become a mainstay for treatment of many oncological diseases, including malignant melanoma. While enhancing anti-tumor immune response, these treatments can also cause autoimmune side effects by promoting self-antigen driven over-activity of the immune system. Special caution is required in treating cancer patients with pre-existing autoimmune diseases. Here, we describe a case in which treatment of malignant melanoma with BRAF/MEK inhibitors led to radiological exacerbation of pre-existing multiple sclerosis (MS). A timeline of events is shown in **Figure 1A**. By in depth-analysis of immune cells both during exacerbation and after successful treatment, we aimed to advance our understanding of balancing immunomodulatory interventions.

## Case description

In February 2022, a 47-year-old female patient presented at the neurological clinic of the University hospital in Mainz, Germany. The patient had been diagnosed with relapsing-remitting MS in 2009. Disease-modifying treatment was initiated with glatiramer acetate and escalated to dimethyl fumarate in 2012, after which the patient had a stable disease course without relapses or disease progression. In June 2020, malignant melanoma was diagnosed located on the right lower leg (Breslow tumor thickness 1.3 mm, not ulcerated, Clark’s level IV, pT2a pN2a M1, stage IV according to 2017 AJCC classification). After resection of tumor and lymph nodes, anti-tumor medication was started in September 2020 with a combination therapy of BRAF inhibitor dabrafenib and MEK inhibitor trametinib. In December 2021, a routine MRI scan showed an exacerbation of radiological disease activity with ten new and gadolinium-enhancing lesions (**Figure 1B**). There were no new focal neurological deficits in clinical examination. At this time point, levels of neurofilament light chain (NfL) in serum were 94.4 pg/ml, indicating axonal damage.

Serological analyses, including antinuclear antibodies (ANA), anti-neutrophil cytoplasmic antibodies (ANCA), anti-dsDNA antibodies, rheumatoid factor, soluble interleukin (IL)-2 receptor, anti-cardiolopin IgG and IgM, β2-glycoprotein I IgG and IgM, myelin oligodendrocyte glycoprotein (MOG) antibodies, anti-aquaporin-4 antibodies, screening for HIV, hepatitis B and C, cryptococcus, Lyme borreliosis and syphilis revealed no evidence of other autoimmune or infectious disease. Analysis of cerebrospinal fluid (CSF) showed a mild lymphocytic pleocytosis with positive oligoclonal bands without evidence for atypical or malignant cells.

**Figure 2:**
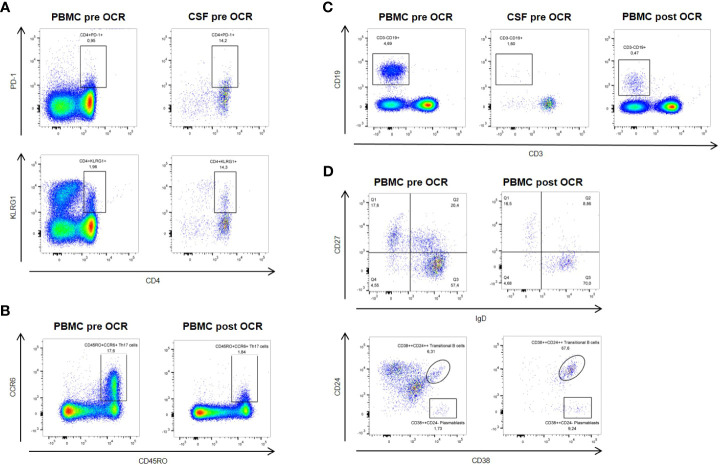
Flow cytometric analysis pre- and post-ocrelizumab (OCR) treatment. **(A)** Activation markers PD-1 and KLRG1 are elevated on CSF compared to peripheral blood CD4+ T cells at baseline. **(B)** Six months after ocrelizumab treatment, CD45RO+CCR6+ Th17 cells are reduced compared to baseline. **(C)** Levels of B cells are reduced under ocrelizumab treatment. **(D)** Ocrelizumab changes B cell subset composition, with decrease of IgD+CD27+ non-switched memory B cells and proportional increase of CD38++CD24++ transitional B cells and CD38++CD24- plasmablasts.

In order to investigate the patient’s immune compartment in more detail, we performed flow cytometry analysis (FACS) for both peripheral blood mononuclear cells (PBMC) and CSF (**Figure 2**). CD4+ T cells composed the largest fraction of lymphocytes (PBMC: 58.9%; CSF: 78.9%) followed by CD8+ T cells (PBMC: 14.1%; CSF: 13.0%) and B cells (PBMC: 4.69%; CSF: 1.6%). Among CD4+ T cells, the fraction of CD45RO+CCR6+ Th17 cells was 17.6% in PBMC (CSF: 52.4%) and the fraction of FoxP3+ regulatory T cells was 0.83% (CSF: 9.61%). Activation markers PD-1 and KLRG1 (on both CD4+ and CD8+ T cells) and CD69 (on CD4+ T cells) were elevated in CSF compared to PBMC. In B cells, expression of the activation marker CD86 was also enhanced in CSF (70.0% compared to 6.18% in PBMC).

Due to the large number of new contrast-enhancing lesions, we decided to treat the patient with 500 mg methylprednisolone i.v. for three days, and then escalate disease-modifying therapy for MS to the B cell-depleting antibody ocrelizumab. The patient received the first cycle of ocrelizumab in February 2022. Oncologic treatment with BRAF/MEK inhibitors was continued. Follow-up in September 2022 showed no signs for MS disease activity, and oncologic follow-ups were also stable. In an MRI scan in January 2023 (**Figure 1B**), no new MS lesions were observed. Levels of NfL in serum were reduced to 11.6 pg/ml.

FACS analysis of PBMC in September 2022 showed a marked reduction of B cells under treatment with ocrelizumab (0.47% of lymphocytes). Subset analysis of B cells revealed that IgD+CD27+ non-switched memory B cells were decreased (8.86% compared to 20.4%) while CD38++CD24++ transitional B cells were strongly increased (67.6% compared to 6.31%), as were CD38++CD24- plasmablasts (9.24% compared to 1.73%). Furthermore, there was a reduction of CD45RO+CCR6+ Th17 cells (1.84% of CD3+CD4+ cells compared to 17.6%) and an increase in FoxP3+ regulatory T cells (2.29% of CD3+CD4+ cells compared to 0.83%). We also observed a slight decrease of activation markers PD-1 and KLRG1 on both CD4+ and CD8+ T cells.

## Discussion

About a decade ago, the introduction of targeted treatments greatly improved prognosis for patients with malignant melanoma. Specifically, inhibitors target the protein kinase BRAF, which is often affected by an oncogenic driver mutation leading to constitutive activation of the MAPK/ERK pathway with enhanced cell proliferation and survival (1). Standard treatment regimens combine BRAF inhibitors with inhibitors of the downstream substrate kinase MEK, further improving progression-free survival (2). Inhibition of this signaling cascade favorably impacts the tumor microenvironment by increase of tumor infiltrating lymphocytes, a shift in T cell subsets and cytokines from immunosuppressive towards cytotoxic, and up-regulation of MHC-expression on tumor cells (3, 4).

However, expression of BRAF and MEK is not restricted to tumor cells. In fact, inhibition of BRAF in wild-type cells without mutation causes paradoxical activation of the MAPK/ERK pathway by promoting transactivation of RAF dimers (5). For example, the BRAF inhibitor vemurafenib has been shown to increase cytotoxic activity and cytokine secretion by T cells (6). Similar results have been described for natural killer (NK) cells and macrophages (7, 8). While these mechanisms are beneficial in driving anti-tumor immune response, over-activation of the immune system might also be detrimental in terms of autoimmune side effects. Accordingly, there have been case reports of inflammatory demyelinating sensorimotor polyradiculoneuropathy (9), sarcoidosis (10), granulomatosis with polyangiitis (11) and also one previous case of MS exacerbation (12), in which BRAF/MEK treatment was subsequently discontinued. Similar effects on autoimmune central nervous system (CNS) diseases might be caused by other anti-tumor treatments such as immune checkpoint inhibitors (13).

Chronic autoimmune inflammation in MS is driven by both T and B cells, and ultimately leads to cumulating axonal damage and neurodegeneration already in young adults (14). During exacerbation under BRAF/MEK treatment in our patient, we observed expression of activation markers on peripheral immune cells that was further enhanced in CSF. In order to effectively treat the MS exacerbation without completely abolishing the anti-tumor immune response, we chose therapeutic B cell depletion by the humanized anti-CD20 antibody ocrelizumab. As expected, FACS analysis showed a significant decrease of B cells in peripheral blood even six months after the first treatment cycle. However, further analysis revealed that different B cell subsets were affected differentially by ocrelizumab treatment (15). More specifically, there was a proportional increase in transitional B cells and plasmablasts. While plasmablasts escape anti-CD20 directed depletion, the relative increase in transitional B cells probably reflect an anti-inflammatory remodelling of the repopulating B cell profile. Interestingly, depletion of B cells also markedly reduced the percentage of circulating Th17 cells, a finding in line with previous reports from B cell-depleting treatment with rituximab in rheumatoid arthritis (16), and enhanced the fraction of regulatory CD4+ T cells. Under continuation of BRAF/MEK therapy, expression of activation markers was only slightly affected by B cell depletion.

**Figure 3:**
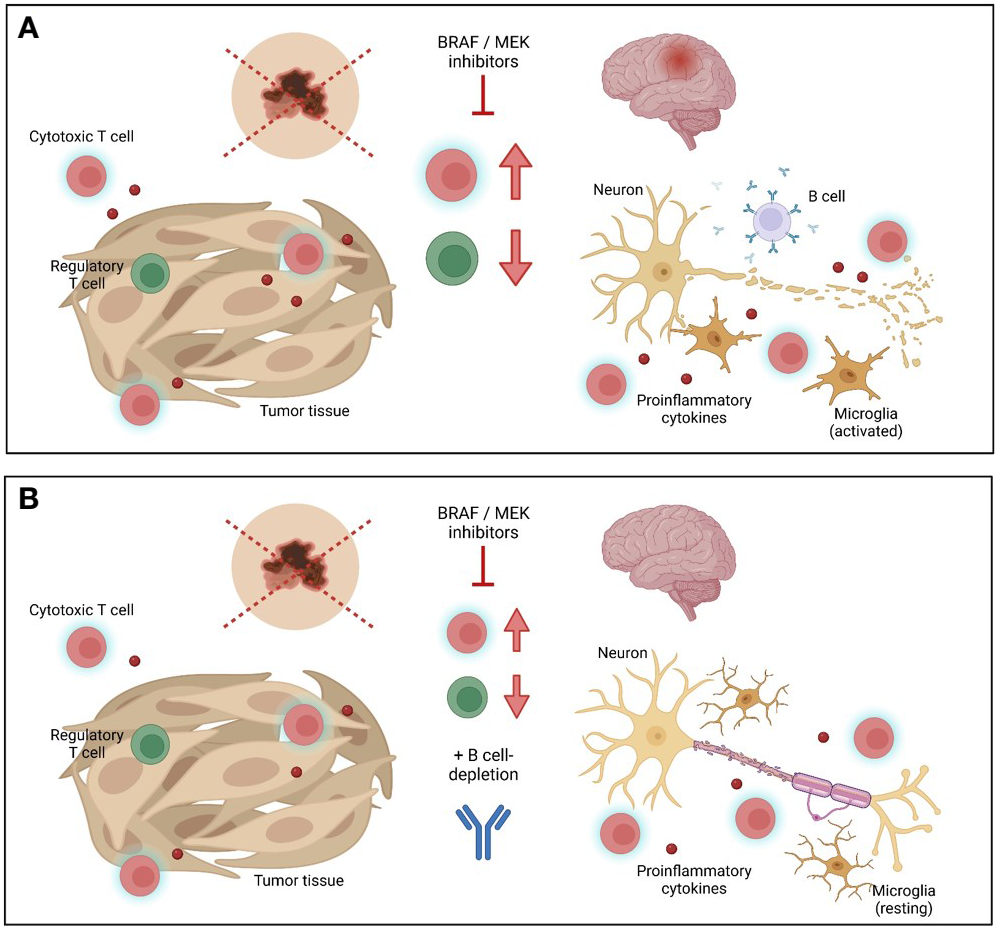
Schematic overview of combined tumor and multiple sclerosis treatment. **(A)** Inhibition of BRAF/MEK leads to beneficial anti-tumor immune response, but may drive autoimmune inflammation in the central nervous system (CNS) in multiple sclerosis (MS) patients. **(B)** Additional treatment with the anti-CD20 antibody ocrelizumab might favorably balance detrimental autoimmunity in CNS while preserving anti-tumor immunity. Graphic was created with Biorender.com.

In sum, targeted anti-tumor treatment can induce autoimmune side effects, an impact of special importance in patients with pre-existing autoimmune conditions. Balancing favorable anti-tumor immune response and potentially devastating exacerbation of autoimmune disease is a major challenge for treating physicians. In our case report, additional targeting of B cells served as a game changer for stabilization of MS, allowing for continuation of anti-tumor treatment (**Figure 3**). Although a larger number of cases and longer time of follow-up are needed in order to provide general recommendations, this case report highlights the possibilities of sequential modulation of immune cell subsets and their surveillance by flow cytometric analysis paving the way for individualized therapeutic concepts.

## Data availability statement

The original contributions presented in the study are included in the article. Further inquiries can be directed to the corresponding author.

## Ethics statement

This study was approved by the Landesärztekammer Rheinland-Pfalz, ethics vote n° 2020-15206_1, and conducted in accordance with the local legislation and institutional requirements. The participant provided her written informed consent to participate in the study and and consented to publication of the data included in this article.

## Author contributions

KP: Conceptualization, Formal Analysis, Investigation, Methodology, Writing – original draft. MP: Conceptualization, Methodology, Writing – review & editing. MS: Conceptualization, Methodology, Writing – review & editing. FS: Formal Analysis, Investigation, Writing – review & editing. FZ: Funding acquisition, Writing – review & editing. SB: Conceptualization, Funding acquisition, Methodology, Writing – review & editing.
